# Reduced susceptibility to selected synthetic pyrethroids in urban malaria vector *Anopheles stephensi*: a case study in Mangalore city, South India

**DOI:** 10.1186/1475-2875-9-179

**Published:** 2010-06-23

**Authors:** Satyanarayan Tiwari, Susanta K Ghosh, Vijay P Ojha, Aditya P Dash, Kamaraju Raghavendra

**Affiliations:** 1 National Institute of Malaria Research, ICMR Complex, Poojanahalli, Kannamangala Post, Devanahalli, Bangalore, 562110, Karnataka, India; 2 National Institute of Malaria Research, Sector 8, Dwarka, New Delhi, 110 077, India; 3 World Health Organization South East Asia Regional Office, Indra Prastha Marg, New Delhi, 110 002, India

## Abstract

**Background:**

Synthetic pyrethroids are potent insecticides most commonly used in the vector control programme. These are applied for indoor residual sprays, space sprays and in impregnated bed nets. Resistance reduces the efficacy of insecticides. Thus, the susceptibility status of the target vector(s) is monitored routinely to select the effective ones. A study was undertaken in a malaria endemic coastal city Mangalore, Karnataka, South India, against the known malaria vector *Anopheles stephensi*.

**Methods:**

The susceptibility status was assessed at diagnostic doses of DDT (4%), malathion (5%), deltamethrin (0.05%), cyfluthrin (0.15%), alphacypermethrin (0.10%), lambdacyhalothrin (0.05%) and permethrin (0.75%) using the standard WHO tube test method during October/November 2006.

**Results:**

*Anopheles stephensi *was resistant to malathion by 54.9%, but tolerant to deltamethrin by 86.1%, cyfluthrin 95.5% and alphacypermethrin 90.6%, whereas it was susceptible to DDT by 98.1%, lambdacyhalothrin 98.6% and permethrin 100.0%, respectively. The KDT_50 _and KDT_95 _values for these insecticides also showed the similar responses.

**Conclusion:**

Susceptibility of *An. stephensi *to DDT is an important finding as this has never been used in Mangalore city, whereas its rural counterpart *Anopheles culicifacies *is widely resistant to this insecticide. The study explores the selection and rotation of the appropriate insecticide molecule even within the same group for effective vector management.

## Background

In recent years, there are many options and strategies to combat diseases transmitted by insect vectors. Insecticides still remain the mainstay of vector control programmes [1]. Synthetic pyrethroids are extensively being used for indoor residual sprays, space sprays and in impregnated bed nets. These are highly effective, if optimally applied, but resistance to these chemicals reduces their impact [2]. Tube and cone bioassays on sprayed surfaces and substrates to monitor the susceptibility status of the target vector(s) are effective tools to assess the efficacy of insecticides. The present work was carried out in Mangalore city, where malaria has been a serious public health problem for more than one and half decades [3]. The vector control strategies adopted to curtail malaria transmission in urban settings are anti-larval operations, whereas indoor residual spraying (IRS) is not recommended. However, personal protection measure with insecticide-treated nets (ITNs), especially with synthetic pyrethroids, has been advocated [4].

Recently under the World Bank-aided Enhanced Malaria Control Programme (EMCP), ITNs have been advocated to control malaria in rural areas. To assess the operational feasibility of ITNs in malaria control in urban areas, a study on long-lasting insecticide-treated nets (LLINs) with alphacypermethrin was carried out in the city from October 2006 to August 2007. Baseline data on susceptibility status of *Anopheles stephensi *to different insecticides were collected prior to the LLIN trial to explore an appropriate insecticide molecule.

## Methods

### Study area

Mangalore city (12°55' LN 74°47'LE, average elevation of 22 m asl) is situated on the Western coast of Arabian Sea in Karnataka, South India. It has 494,629 inhabitants living in an area of 132.45 km^2^. The climate is tropical with dry season (January-May), monsoon (June-August) and post-monsoon (September-December). Average temperature ranges between 16 and 35°C, average relative humidity around 85%, and 4400 mm of average annual rainfall. It is a port city with many activities that attract labour migrants (8-12% of urban population) engaged in construction related works. The recorded literacy rate is 83%. The city has 60 municipal wards, and the health services are provided by five municipal corporation hospitals and two government hospitals, besides five local medical colleges. In this city, the annual parasite incidence (API; cases per 1,000 population annually) varies from 1.2 to 3.1, and contributes nearly 15-20% of the total malaria cases with few deaths annually in Karnataka, and nearly 0.2-0.8% in India [3]. *Anopheles stephensi *is the main malaria vector that preferably breeds in wells, over head tanks, cement tanks, fountains and also in masonry tanks, curing waters in construction sites [5]. Active transmission is recorded during the post-monsoon months, and recently this insect vector has been incriminated in this city [5]. The vector control strategies adopted to curtail malaria transmission are anti-larval operations using chemical larvicides mainly temephos and fenthion (both organophosphorus compounds), indoor pyrethrum spray, outdoor fogging with malathion and synthetic pyrethroids especially deltamethrin and cyfluthrin. Larvivorous fish in wells, fountains, water storage cement tanks, masonry water tanks at construction sites, are additionally applied.

### Insecticide susceptibility tests

**Figure 1:**
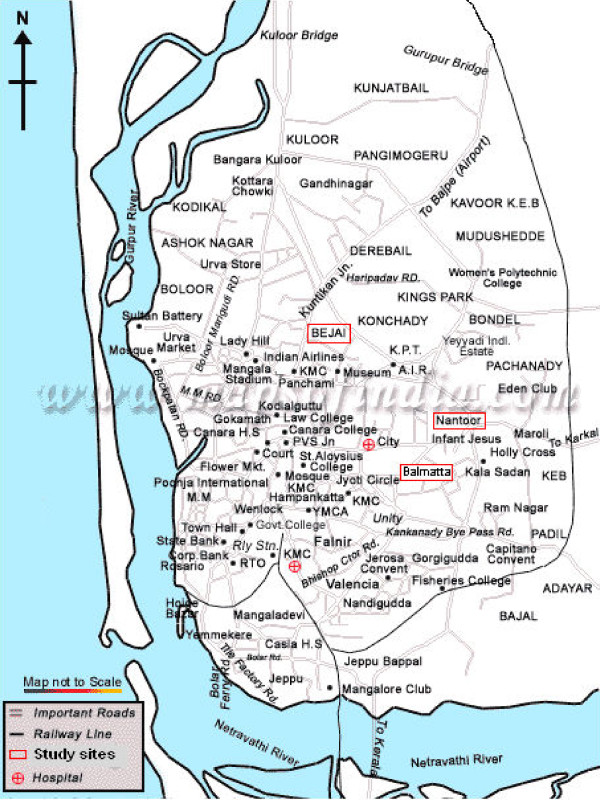
**Map showing the study sites in Mangalore City, Karnataka, South India**.

Susceptibility of insecticides by tube test method was carried out according to the standard WHO procedure during October/November 2006 [6-8] with WHO-supplied insecticide-impregnated papers as per diagnostic doses, DDT (4%), malathion (5%), deltamethrin (0.05%), cyfluthrin (0.15%), alphacypermethrin (0.10%), lambdacyhalothrin (0.05%) and permethrin (0.75%). Immature stages of anopheles mosquitoes collected from three sites, Nanthoor, Bejai and Balmatta in the city (Figure 1), brought live to the laboratory, reared to adults. Emerged *An. stephensi *females were fed on mice blood and full-fed mosquitoes were exposed to the insecticide-impregnated papers for 1 h. Experiments were conducted with a minimum of three replicates with matched controls for each insecticide in a room maintaining temperature at 27 ± 2°C and 80% relative humidity. The knockdown effect of each insecticide was recorded every 10 minutes over the 1-h exposure period. Exposed mosquitoes were then transferred to a recovery tube and provided with 10% glucose solution. Final mortality was recorded after 24 h of holding time in experimental and control tubes. Mortality in control between 5 and 20% was corrected with Abbott's formula [9] and expressed as corrected percent mortality.

### Data analysis

Data were analysed using VassarStats software [10]. Fifty and 95% knockdown times (KDT_50 _and KDT_95_) were estimated by means of a log-time probit model using the Ldp line^R ^software [11,12].

## Results

The result of bioassays is summarized in Table 1. As per the WHO criteria, mortality < 80% is considered to be resistant, 80-98% as tolerant (evaluation required) and > 98% as susceptible [6]. Accordingly, *An. stephensi *in Mangalore city was resistant to malathion, having 54.9% (95% CI: 46.74-72.42) mortality, but susceptible to DDT, lambdacyhalothrin and permethrin showing mortalities of 98.1% (95% CI: 92.95-103.19), 98.6% (95% CI: 94.10-103.05) and 100% respectively. Mortalities recorded to deltamethrin, cyfluthrin and alphacypermethrin were 86.1% (95% CI: 76.38-103.93), 95.5% (95% CI: 79.00-110.94) and 90.6% (95% CI: 85.34-95.90), respectively, indicating tolerance.

**Table 1:** Susceptibility status of *An. stephensi *(October/November, 2006) to standard doses of insecticides (7 such) following WHO methods* (1975) in Mangalore city, Karnataka, South India

Diagnostic insecticide dose on impregnated papers^#^	Mosquitoes exposed (n)		Dead mosquitoes after 24 h of exposure (n)		Mortality after 24 h of exposure (%)		Corrected mortality (%)^§^	KDT_50 _(CL)	KDT_95 _(CL)
	Expt	Cont	Expt	Cont	Expt (95% CI)	Cont (95% CI)			
DDT 4%	60	60	59	7	98.3 (92.99-103.65)	11.65 (6.33-16.89)	98.1 (92.95-103.19)	19.24 (18.87-21.48)	55.67 (47.78-68.50)
Malathion 5%	106	106	65	15	45.36 (13.98-76.74)	14.06 (10.93-17.19)	54.9 (46.74-72.42)	765.82^‡^	34147.10^‡^
Deltamethrin 0.05%	72	72	63	7	86.62 (69.78-103.46)	9.62 (6.24-3.00)	86.1 (76.38-103.93)	37.33 (35.02-39.88)	72.73 (64.96-85.11)
Cyfluthrin 0.15%	77	57	74	8	95.57 (78.5-109.64)	13.93 (9.35-18.51)	95.5 (79.00-110.94)	31.74 (29.50-33.96	69.74 (62.03-81.56)
Alpha-cypermethrin 10%	68	50	62	3	91.2 (86.27-96.13)	6.03 (4.52-7.54)	90.6 (85.34-95.90)	30.33 (27.69-33.12)	83.26 (70.79-104.26)
Lambda-cyhalothrin 0.05%	80	60	79	5	98.75 (94.78-102.72)	8.33 (1.17-15.49)	98.6 (94.10-103.05)	19.20 (17.34-20.97)	50.42 (44.99-58.17)
Permethrin 0.75%	77	57	77	3	100.0 (100.0-100.0)	5.3 (4.04-6.59)	100.0 (100.0-100.0)	16.83 (14.87-18.67)	47.93 (41.35-58.52)

* Mortality recorded < 80% considered resistant, 80-98% tolerant (evaluation required), while > 98% as sensitive.

^#^Supplied by WHO.

^§^Corrected mortality was calculated following Abott's formula (1925).

^‡^Confidence limit could not be calculated due to large g values.

Exp: Experimental; Cont: Control; CI: Confidence interval; CL: Confidence limit

The knockdown effect determined over a 1-h period indicated that, KDT_50 _and KDT_95 _for malathion was the highest among the seven insecticides tested. These values were also higher for deltamethrin, cyfluthrin and alphacypermethrin than DDT, lambdacyhalothrin and permethrin (Table 1).

## Discussion

Resistance to both DDT and malathion in *An. stephensi *was reported earlier from the rural area of Harayana [13], from urban areas of Goa [14], and Delhi [15]. In Bikaner district of Rajasthan, it was reported resistant to DDT and tolerant to both malathion and permethrin [16]. The present study showed that *An. stephensi *is susceptible to DDT and permethrin, whereas it is resistant to malathion. The KDT_50 _and KDT_95 _values were also maximum for malathion and minimum for permethrin, suggesting that the knockdown resistance mechanism could be operating in the mosquito population, as has been demonstrated in lab in *An. stephensi *with deltamethrin [17].

In Mangalore city, the possible reason for the development of resistance in *An. stephensi *to malathion and low mortalities in deltamethrin and cyfluthrin could be attributed to selective pressure from fogging, IRS including anti-larval operations with these molecules for more than a decade. Tolerance in alphacypermethrin could be the consequence of cross-resistance among synthetic pyrethroid molecules exhibiting similar resistance mechanism [17,18].

## Conclusion

Susceptibility of *An. stephensi *to DDT is an important finding, whereas its rural counterpart *Anopheles culicifacies *is widely resistant to this insecticide [18]. The reason could be that this molecule has never been used in Mangalore city [3], thus maintaining a status of susceptibility in the area in absence of selection pressure.

In the ITN programme, only synthetic pyrethroids are being used for impregnation of nets. A recent report indicated the evolution of resistance in *An. gambiae *to pyrethroids in Africa [2], and the reduced susceptibility of *An. stephensi *to deltamethrin, cyfluthrin and alphacypermethrin in Mangalore city is a matter of concern. In view of the current scenario, emphasis could be given on other methods of vector control, preferably biocontrol with larvivorous fish and different formulations of biolarvicides especially *Bacillus thuringiensis *var. *israelensis *under the integrated vector management programme [19,20]. In Mangalore city, open dug wells are present in the courtyard almost in every house and these serve as major foci (ecological niche) for *An. stephensi*. The construction activities begin in the vicinity of such wells leads to profuse breeding of *An. stephensi *in the curing waters, masonry tanks and other water collection created during the activities. Immigrant labourers from malaria endemic areas act as reservoirs add to the problem immensely who reside near to these sites. The current application of fogging should be discontinued as these are not recommended for routine vector control programme [4]. Thus the malaria control operation in the city should focus alternatively on anti-larval strategies [4]. Fortnightly application of biolarvicides in the curing waters in the construction areas and introduction of larvivorous fish particularly *Poecilia *[19,20] in all the breeding habitats especially in wells would be the best option for anti-larval measures. Integrated vector management strategies specific to this urban setting need to be optimally used for its appropriate implementation.

## Competing interests

The authors declare that they have no competing interests.

## Authors' contributions

ST, SG and KR conceived and arranged the entire study. VPO assisted in conduction of the test in the field. APD reviewed and edited the paper. All authors helped write, read and approved the final manuscript.
